# Redefining the Role of Chemotherapy and Targeted Cytotoxic Delivery in Lung Neuroendocrine Tumors

**DOI:** 10.1111/1759-7714.70376

**Published:** 2026-08-13

**Authors:** Alice Laffi, Giuseppe Lamberti, Anna La Salvia

**Affiliations:** ^1^ Humanitas Gavazzeni, Medical Oncology Bergamo Italy; ^2^ Department of Medical and Surgical Sciences (DIMEC) Alma Mater Studiorum ‐ University of Bologna Bologna Italy; ^3^ National Center for Drug Research and Evaluation, National Institute of Health (ISS) Rome Italy

**Keywords:** chemotherapy, lung carcinoids, lung NETs, neuroendocrine tumors

## Abstract

The clinicopathological and therapeutic landscape of lung neuroendocrine tumors (NETs) is continuously evolving. Although systemic chemotherapy has historically represented a management cornerstone, the available data are predominantly retrospective and outdated. A critical review of the current literature and public clinical trial registries was performed to evaluate the role of chemotherapy and targeted cytotoxic delivery systems in lung NETs. Given the nature of the review, the evidence was synthesized descriptively. In early‐stage disease, the clinical utility of perioperative (neoadjuvant and adjuvant) chemotherapy remains uncertain and lacks universal standardization. In the advanced or metastatic setting, traditional platinum/etoposide regimens demonstrate suboptimal efficacy in controlling well‐differentiated carcinoids. Conversely, oral alkylating agents (temozolomide) and combination schedules such as CAPTEM (capecitabine/temozolomide) or temozolomide paired with cabozantinib offer promising response and disease control rates. Future strategies are moving away from unselected systemic cytotoxicity toward targeted delivery and chemo‐immunotherapy platforms, leveraging peptide‐drug conjugates (PDCs like PEN‐221), antibody‐drug conjugates (ADCs), and bispecific T‐cell engagers (BiTEs) directed against emerging surface targets such as DLL3 (e.g., tarlatamab) and TROP2. There is an urgent, unmet clinical need for prospective, multicenter clinical trials and international registries. Dissecting the complex molecular landscape of lung NETs will be essential to identify predictive biomarkers and actionable therapeutic targets, ultimately transitioning clinical management from empirical, extrapolated choices to robust, evidence‐based, stage‐specific standards of care.

## Graphical abstract

Perioperative chemotherapy utility remains uncertain and lacks universal standardization. Platinum/etoposide shows suboptimal efficacy in lung NET (neuroendocrine tumors); oral alkylators (temozolomide) alone or in combination (e.g., with capecitabine), and cabozantinib offer promising disease control. Transition toward targeted platforms including peptide‐drug conjugates (PDCs like PEN‐221), antibody‐drug conjugates (ADCs), and bispecific T‐cell engagers (BiTEs like tarlatamab) against targets like DLL3 and TROP2.

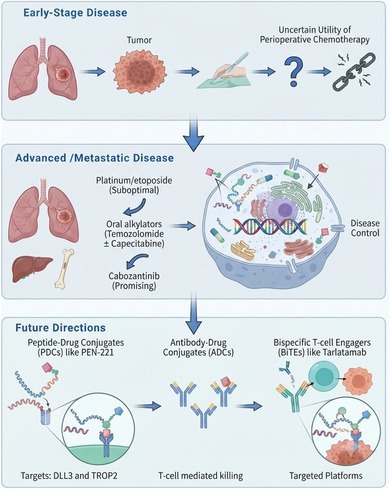

## 1 Background and Rationale

Neuroendocrine tumors (NETs) of the lung represent a heterogeneous group of malignancies that are continuously evolving in terms of classification [1, 2]. A major milestone was reached with the 1999 World Health Organization (WHO) classification which standardized lung NETs into four distinct subcategories—typical carcinoid, atypical carcinoid, large cell neuroendocrine carcinoma (LCNEC), and small cell lung cancer (SCLC) [3]. This categorization is primarily based on the proliferation index, expressed as MIB‐1 or Ki‐67, and morphological features; carcinoids/NETs are well‐differentiated with a low proliferation index, whereas NECs are poorly differentiated and exhibit a high proliferation index. Currently, the field is transitioning toward the integration of novel categories, including high‐proliferative lung NETs and combined neuroendocrine/non‐neuroendocrine entities. More recently, the WHO 2021/2022 classification update has emphasized the incorporation of immunohistochemical and molecular features into diagnostic algorithms, while also recognizing emerging entities within the pulmonary neuroendocrine neoplasm (NEN) spectrum. In particular, increasing attention has been directed toward high‐proliferative carcinoid tumors characterized by elevated mitotic counts and/or Ki‐67 proliferation indices, which appear biologically intermediate between well‐differentiated carcinoids and NECs [4]. Although these lesions are not yet formally recognized as a distinct category, they are acknowledged as an evolving entity requiring further clinical and molecular characterization. Furthermore, the current classification recognizes combined neuroendocrine/non‐neuroendocrine neoplasms and highlights the growing relevance of molecular profiling in refining prognostic stratification and therapeutic decision‐making [5, 6]. This paradigm shift aims to align the thoracic classification more closely with the gastro‐entero‐pancreatic (GEP‐NET) framework [4]. More recently, updated guidelines from ENETS and IASLC have adopted a grade‐based classification system, mirroring that of GEP‐NENs [7, 8]. Whit this new nomenclature, typical and atypical carcinoids are redefined as grade (G) 1 and 2 NETs, while high‐proliferative carcinoids are now defined as G3 NETs.

Parallel to this evolving pathological landscape, the therapeutic scenario has also undergone significant changes with the introduction of targeted therapies and radioligand treatment (RLT). However, among targeted agents, everolimus remains the only approved targeted therapy for advanced progressive lung NETs, based on the results of the phase III RADIANT‐4 trial [9] and its dedicated lung‐subgroup analysis [10]. In parallel, RLT with [177Lu]Lu‐DOTATATE has emerged as a promising therapeutic strategy in somatostatin receptor‐positive NENs; nevertheless, unlike GEP‐NETs, RLT is not currently formally approved for pulmonary NETs, and evidence in this setting mainly derives from retrospective series and subgroup analyses [11, 12]. Available data suggest encouraging antitumor activity and a manageable safety profile in selected patients with advanced pulmonary NETs, although prospective studies are still lacking. Historically, systemic chemotherapy has been the cornerstone of management for lung NECs and, under specific clinical conditions, for advanced or metastatic lung NETs. However, the available data endorsing chemotherapy in lung NETs are predominantly retrospective and outdated.

Consequently, the guidelines themselves are based on low level of evidence, resulting in discordant recommendations. For instance, while ENETS (European Neuroendocrine Tumors Society) guidelines indicate chemotherapy primarily for palliative purposes [7, 13], NANETS (The North American Neuroendocrine Tumor Society) [14] and ESMO (European Society of Medical Oncology) [15] guidelines suggest it may have a role in the adjuvant setting, beyond its use in metastatic disease.

Recently, an international survey conducted by the ENETS Lung NET Task Force highlighted substantial heterogeneity in clinical practice, particularly in the management of early‐stage disease. The survey demonstrated considerable variability in adjuvant treatment and follow‐up strategies, even among ENETS Centers of Excellence, emphasizing the current lack of consensus and the urgent need for prospective studies to support evidence‐based recommendations [16].

## 2 Methods

We conducted a critical review of the current literature regarding the use of chemotherapy and targeted cytotoxic delivery systems in lung NETs. To ensure methodological transparency, a comprehensive literature search was performed in the PubMed database using keywords such as “lung carcinoids,” “Lung NETs,” “chemotherapy,” “cytotoxic,” “neoadjuvant,” and “adjuvant”. We selected only articles published in the English language between January 2000 and December 2025. Furthermore, we integrated our findings with evidence‐based recommendations from international guidelines on the topic (ENETS and ESMO), and identified ongoing clinical trials via public registries. Given the critical nature of the present, the available evidence was reported descriptively. This review aims to analyze the current evidence supporting chemotherapy in lung NETs, underlining the urgent, unmet clinical need for prospective, multicenter clinical trials.

## 3 Results

### 3.1 Chemotherapy in Lung Carcinoids/NETs: Current Clinical Evidence

In contrast to the clear‐cohered therapeutic algorithms established for lung NECs, the clinical role of chemotherapy in pulmonary NETs remains heterogeneous and relies on low‐quality evidence that still precludes guidelines from endorsing precise chemotherapeutic regimens [1, 2, 5, 7, 13]. Historically relegated to retrospective observations, cytotoxic strategies are currently being reevaluated across different stages of the disease. In the early‐stage setting, systemic treatment is navigating significant uncertainties; its clinical utility is currently under scrutiny both as a neoadjuvant tool aimed at local downstaging and tumor shrinkage to enable radical surgery and as an adjuvant strategy to mitigate the long‐term risk of recurrence in poorly defined high‐risk populations. Conversely, in the advanced or metastatic setting, the therapeutic paradigm is rapidly shifting. While standard platinum regimens are finally deprioritized due to suboptimal response rates in carcinoids/lung NETs, alternative schedules—particularly oral alkylating agents and targeted therapies—are emerging as the modern backbone of disease control.

### 3.2 Chemotherapy in Lung Carcinoids/NETs: Is There a Neoadjuvant Role?

Differently from the established therapeutic protocols for poorly‐differentiated lung NENs [17], and as highlighted by the ENETS survey, the role of systemic treatment for early‐stage lung NETs remains uncertain and lacks universal standardization [16]. While surgical resection is the cornerstone of management for localized disease, a little effort has been made to clarify whether or not there is a role for perioperative therapy.

In 2021, the NIKE (Neuroendocrine Tumors, Innovation in Knowledge and Education) group reviewed existing literature on neoadjuvant treatments for lung NENs [18]. However, the most of collected studies were case series focusing on LCNEC or SCLC (small cell neuroendocrine carcinoma), and the outcomes were inconsistent against not standardized therapeutic approaches. Moreover, in these studies, it remains to be questioned how many cases of LCNEC, if reevaluated according to the current histopathologic classification, would be identified as high‐proliferative atypical carcinoids/NETs. In literature, there are few cases on the role of neoadjuvant therapy on lung NET. The report by Evangelou et al. [19] showed a response rate similar to what found for metastatic disease to CAPTEM (capecitabine + temozolomide) regimen, consenting the patients to be submitted to the radical surgery. Another old analysis of 45 patients by Srirajaskanthan et al. [20] reported a good response to a preoperative chemotherapy with 5‐fluoururacil, cisplatin and streptozocin but the histopathologic features of the cases were not specified. On the same line, Turner et al. reported the findings on 79 patients with advanced or metastatic NETs treated with 5‐FU, cisplatin and streptozocin. Although any conclusion can be drawn for the paper proposal, one patient with locally advanced, not operable lung NET experienced a partial response that enable surgery of primary and nodal disease [21].

Within the broader field of NETs, the concepts of neoadjuvant or perioperative intervention have been insufficiently explored, largely due to the indolent biological behavior of these tumors. Pancreatic NETs, as usual, have served as the benchmark for evaluating neoadjuvant strategies [22, 23]. In this context, chemotherapy is the most common treatment proposed [24]; furthermore, the last decade has seen the emergence of RLT as a promising neoadjuvant modality [25].

The main aims of neoadjuvant therapy are to enhance the surgical resectability, through a local downstaging—targeting both the primary lesion and involved lymph nodes—while simultaneously reducing the risk of distant spread. When considering prognostic indicators at the time of diagnosis, excluding non‐modifiable factors such as histotype (e.g., atypical carcinoid), both a resection margin of less than 2 cm and the presence of nodal involvement in surgical specimens have been identified as significant negative predictors for recurrence [26]. To date, investigations into recurrence rates remain limited and predominantly retrospective, lacking a comprehensive, prospective evaluation of the therapeutic impact of preoperative interventions in lung NETs [27, 28].

Given that the 5‐ and 10‐year recurrence rate for atypical carcinoids/NETs G2 are estimated at 18% and 32%, respectively, the NET‐dedicated scientific community must prioritize a multidisciplinary effort to rigorously evaluate the clinical impact of preoperative strategies in lung NETs. Despite the relatively indolent biology of many well‐differentiated lung NETs, the substantial risk of long‐term relapse observed in selected patients, particularly those with atypical histology, nodal involvement, larger tumors, or elevated proliferative indices, highlights the urgent need for more effective risk‐adapted treatment approaches beyond surgery alone. Ultimately, the integration of histopathological, molecular, and functional imaging biomarkers into prospective clinical trials will be essential to identify patients at higher risk of recurrence who may derive meaningful benefit from systemic treatment in the perioperative setting, while avoiding overtreatment in individuals with more indolent disease behavior.

### 3.3 Chemotherapy in Lung Carcinoids/NETs: Is There an Adjuvant Role?

Given the dearth of evidence in the preoperative setting, international guidelines briefly address adjuvant approaches, reserving exclusively for high‐risk lung NETs [7, 13, 14, 15].

Retrospective analyses on the topic have generally reported no survival benefit; nevertheless, adjuvant treatment—comprising chemotherapy and/or radiotherapy—is often suggested for consideration in fit patients with a high risk of relapse. However, no trials have yet been designed to investigate the role of adjuvant treatment in lung NETs, particularly for high‐risk cohorts, the definition of which remains ill‐defined. The limited retrospective data available even suggests a worse prognosis for patients treated with adjuvant chemotherapy, and no definitive conclusions can be drawn regarding the role of radiotherapy in *N*+ lung NETs [29, 30, 31].

The primary challenges in establishing a perioperative treatment framework consist in (i) defining the high‐risk criteria for lung NETs, (ii) selecting the most appropriate systemic therapy regimen, and (iii) determining the optimal number of cycles (or duration) for each phase of the perioperative approach. As presented during the 2023 ENETS conference regarding pancreatic NETs, guided by data extrapolated from the metastatic setting, an expert consensus and formal recommendations should be established to shape the design of future studies. Similarly to other rare diseases, such as gastrointestinal stromal tumor (GIST), where the management of high‐risk neoplasms has evolved significantly over the years—from 1 to 3 and now potentially to 6 years of adjuvant therapy [32, 33, 34, 35], a similar paradigm could potentially be adopted for lung NETs as well. Given the characteristic long‐term disease‐free survival (DFS) following surgery and the lack of standardized protocols for the cessation of follow‐up, adjuvant therapy may play the key role of prolonging the time to recurrence and delaying the onset of distant metastases.

In this framework, the risk–benefit profile of the proposed treatment must be carefully considered, accounting for short‐ and long‐term risks—including toxicity and impact on quality of life (QoL)—against the expected clinical benefits.

### 3.4 Chemotherapy in Lung Carcinoids/NETs: Therapeutic Proposals in Advanced Disease

Historically, the combination of platinum/etoposide served as the standard of care for all metastatic high‐grade lung NECs, yielding a RR ranging between 8% and 23%, and a median PFS of approximately 8 months. On the other hand, the adoption of the same regimens for well‐differentiated lung NETs has been analyzed in several retrospective studies. Based on these, international guidelines propose platinum/etoposide regimen for lung NETs characterized by an aggressive biologic behavior [9, 10]. According to Grandberg et al. however, only 2 out of 8 patients treated with platinum/etoposide experienced a response, while one achieved stable disease (SD); similar findings were reported for streptozotocin and 5‐FU (fluorouracil) [33]. Wirth et al. reported consistent results, with an RR in 2 out of 12 (16%) receiving platinum/etoposide regimen [36].

Rubino et al. [37] evaluated patients with highly proliferative lung NETs (Ki‐67 > 20%) treated with different systemic regimens. Within the cohort receiving platinum/etoposide, the best observed response was SD in 55% of cases, with a median PFS of 5.1 months (95% CI, 0.7–9.4). However, when compared with targeted therapies such as everolimus, the platinum/etoposide regimen has demonstrated to be less competitive in disease control. Indeed, patients treated with everolimus achieved SD in 100% of cases, with a median PFS of 12.1 months (95% CI, 0.3–24), aligning with the lung‐subgroup data from the RADIANT‐4 trial [10].

Chong et al. [38] also reported their experience with large sample of 300 patients affected by advanced or metastatic lung NETs treated with different regimens. In this study, the platinum/etoposide cohort achieved a 23% RR and a PFS of 7 months, which is consistent with the aforementioned case series.

Consequently, alternative regimens containing alkylating agents have been analyzed.

Within the same study by Chong et al. [38] temozolomide‐based chemotherapy achieved an RR of 14% and a median PFS of 10 months.

A Phase II trial by Fine et al. highlighted the efficacy of CAPTEM (capecitabine/temozolomide) regimen, reporting a promising 41% RR and a median PFS exceeding 22 months [39], similar to the same outcomes achieved with the CAPTEM regimen on gastro‐entero‐pancreatic (GEP) NETs [40].

Rubino et al. also provided data regarding other combination strategies: the combination of fluoropyrimidines and oxaliplatin (FOLFOX) showed a median PFS of 6.8 months (95% CI 0–14.9) [37] whereas alkylating‐based chemotherapy achieved a median PFS of 10.2 months (95% CI 0.4–19.9).

Among other combination regimens, alternative schedules have been proposed (e.g., GEMOX—gemcitabine and oxaliplatin—or streptozocin‐based chemotherapy) [41]. However, specific data regarding the lung cohort were either unavailable or inconclusive due to small sample size [21].

The ATLANT trial [42], specifically dedicated to lung NETs, reported a 9‐month Disease Control Rate (DCR) of 35% using temozolomide in combination with lanreotide, yielding a median PFS of 37.1 weeks.

Another recent combination involving temozolomide and cabozantinib—a multitargeted therapy against MET/AXL/VEGFR—was analyzed in a phase II study by Cives et al. The trial reported a RR of 25% in the lung NET cohort, while a median PFS not reached at the time of the study entry [43].

Lastly, when evaluated as monotherapy, temozolomide showed a RR of 14% and a clinical benefit rate of 66% in an early study by Crona et al. [44], and a median PFS of 5.3 months in pretreated lung NETs in the study by Ekeblad et al. [44].

Current guidelines suggest that objective RRs for chemotherapy in lung NENs range between 16% and 30%. However, a critical disparity remains: while GEP‐NETs management is supported by robust, prospective, multicenter phase III trials, the evidence for lung‐primary NETs is predominantly derived from retrospective series with limited sample sizes. This highlights an urgent need for dedicated, prospective trials to define the optimal sequencing of therapies in this specific population.

### 3.5 Chemotherapy in Lung Carcinoids/NETs: What Lies Ahead?

Driven by the need for more effective options, the future of chemotherapy in metastatic lung NETs is moving away from traditional, unselected systemic cytotoxicity toward targeted cytotoxic delivery and innovative chemo‐immunotherapy combinations. It is important to acknowledge, however, that current therapeutic strategies are largely derived from evidence in SCLC and extrapulmonary NENs. Clinical data specific to lung NETs remain limited, and these novel strategies are currently being extrapolated based on shared molecular targets rather than histology‐specific trials. Rather than abandoning chemotherapy, current prospective studies are redefining its application by utilizing cytotoxic agents as high‐potency payloads within antibody‐drug conjugates (ADCs) or peptide‐drug conjugates, or by combining traditional regimens with novel immune platforms.

A primary example of this evolving landscape is the development of peptide‐drug conjugates that deliver highly potent chemotherapy directly to the tumor site. At the 2018 ASCO (American Society of Clinical Oncology) Johnson et al. reported the results of a phase I study evaluating PEN‐221, a novel peptide‐drug conjugate consisting of a somatostatin analog (SSA) linked to the cytotoxic microtubule inhibitor DM1 (emtansine). The trial enrolled patients with advanced, somatostatin receptors 2 positive (SSTR+) NET (including GEP, lung, pheochromocytoma) as well as small cell lung cancer (SCLC) [45]. While the primary aim was to determine the maximum tolerated dose (established at 18 mg), promising efficacy was observed across the overall cohort. However, the subsequent phase II part of the study, presented at the 2021 ASCO, exclusively enrolled only gastrointestinal patients; thus, despite promising, the findings cannot be evaluated for the topic of the present review [46].

Beyond peptide‐targeted chemotherapy, expanding scientific interest focuses on leveraging cytotoxic payloads through ADCs, bispecific T‐cell engagers (BiTEs), and the identification of novel therapeutic targets to guide chemo‐immune strategies. DLL3 (delta‐like ligand 3) has emerged as a particularly compelling target in lung malignancies. Tarlatamab [47], a first‐in‐class anti‐DDL3 BiTE, has already received Food and Drug Administration (FDA) approval for SCLC and is currently undergoing evaluation in other extrapulmonary NECs [48]. Although histology‐specific trials for lung NETs are not yet active, DLL3 expression has been well documented in both SCLCs and well‐differentiated lung NETs [49, 50, 51, 52], providing a strong rationale for exploring this therapeutic avenue. Notably, Cooper et al. recently reported a promising clinical response to tarlatamab in a female patient with a DLL3‐positive atypical lung carcinoid/NET G2 [53].

Currently, further strategies integrating chemotherapy payloads with targeted and immune agents are being actively investigated under the DLL3 axis. An ongoing phase I/II trial is evaluating the safety of the anti‐DLL3 targeting agent gocatamig, administered either in combination with atezolizumab or with the topoisomerase I inhibitor‐based ADC ifinatamab deruxtecan (I‐DXd)—a novel ADC that utilizes a topoisomerase I inhibitor chemotherapy payload—in high‐grade NENs with elevated DLL3 expression (MK‐6070‐001/HPN328‐4001 trial, NCT04471727). Concurrently, another phase I trial is assessing the safety and preliminary efficacy of ABBV‐706, an ADC targeting SEZ‐6 with a topotecan payload, administered either as monotherapy or combined with budigalimab, carboplatin, or cisplatin, explicitly exploring the synergy between advanced cytotoxic agents and conventional platinum doublets in high‐grade NETs (NCT05599984).

Similarly, TROP2 represents another promising surface antigen for directed chemotherapy delivery via ADC‐based strategies. The TROP2‐directed ADC sacituzumab govitecan, carrying a potent irinotecan‐derived payload, is currently under evaluation in an ongoing trial for progressive, advanced, extrapulmonary high‐grade NENs (SKB264‐II‐NEN, NCT07077564). Prior to this, some authors had already demonstrated encouraging activities of this agent in SCLC and LCNEC [54]. Given that TROP‐2 expression has already been demonstrated across GEP NETs [55], and in lung cancers, including both small‐cell and non‐small‐cell histologies [56], these data collectively establish a solid benchmark for future prospective clinical trials evaluating targeted chemotherapy delivery specifically tailored to lung NETs (Table 1).

**TABLE 1:** Therapeutic strategy and clinical management in lung NET patients.

Clinical setting	Indication	Regimen	Evidence in literature
Neoadjuvant setting	Locally advanced lung NETs	CAPTEM (capecitabine + temozolomide)	– Partial response enabling surgery in case series [18, 19]
		5‐Fluoruracil (5‐FU) + cisplatin + streptozotocin	– Potential response enabling radical surgery in locally advance, inoperable lung NET [20, 21, 22, 23]
Adjuvant setting	Lung NET undergone to radical surgery	Cytotoxic chemotherapy and/or radiotherapy	– No survival benefit or worse prognosis for patients treated with adjuvant chemotherapy [29, 31] – No definitive conclusions can be drawn for radiotherapy in *N*+ disease
Advanced/metastatic setting	Reduction of progression disease and/or secreting activity	Platinum + etoposide	– RR less than 23% – Less competitive compared with other systemic treatments or regimens (e.g., targeted therapies) [34, 35, 36, 37, 38]
		CAPTEM	– RR 41% and PFS > 22 months [39]
		Temozolomide ± lanreotide	– PFS 37.1 weeks [42]
		Temozolomide + cabozantinib	– RR 25% [43] – Median PFS not reached at entry
Advanced/metastatic setting: Peptide‐drug conjugates (PDC)	Advanced SSTR2‐positive NETs and SCLC	PEN‐221 (somatostatin analog + DM1/emtansine payload)	– Phase I study [46]: good tolerability and preliminary efficacy in SSTR2+ NENs
Advanced/metastatic setting: ADCs and BiTEs (DLL3 Axis)	Atypical carcinoids/NET G2 and lung NENs expressing DLL3 High‐grade NENs	Tarlatamab (anti‐DLL3 BiTE) Gocatamig + atezolizumab/ifinatamab deruxtecan (I‐DXd)	– Promising clinical response in a female patient [53] – (NCT04471727): Ongoing Phase I/II trial evaluating the anti‐DLL3 agent gocatamig combined with either atezolizumab or the topoisomerase I inhibitor‐based ADC ifinatamab deruxtecan (I‐DXd)
Advanced/metastatic setting: ADCs (SEZ‐6 and TROP2 Axes)	High‐grade lung/extrapulmonary NENs and NETs SCLC and LCNEC	ABBV‐706 (anti‐SEZ‐6 + topotecan payload) Sacituzumab govitecan/SKB264 (anti‐TROP2 + irinotecan‐derived payload)	– ABBV‐706 (NCT05599984): ongoing phase I trial evaluating this anti‐SEZ‐6 ADC as monotherapy or combined with budigalimab, carboplatin, or cisplatin – Activities of this agent [56]

Nonetheless, these findings must be interpreted with caution; dedicated, histology‐specific clinical trials in lung NETs are urgently required to validate the safety and efficacy of these agents in this specific patient population, as the current clinical landscape for lung NETs still lacks the robust, prospective evidence available for other neuroendocrine malignancies.

## 4 Conclusion

Although chemotherapy represents a crucial tool for lung NETs, considerable effort is still required to define its stage‐specific application. In line with the recommendations of the new ENETS guidelines for the adjuvant setting, there is a strong advocacy for the collection of real‐world data to address all the unmet clinical needs regarding chemotherapy. Within this framework, identifying early predictive biomarkers for high‐risk histotypes or stage, and risk of relapse, recurrence, or progression will be essential to design trials evaluating early therapeutic interventions and optimal treatment sequencing.

To achieve this, the scientific community must first address the critical gaps inherited from the past. Historical evidence is severely limited by small, retrospective cohorts, heterogeneous diagnostic criteria, and the confounding inclusion of poorly differentiated NECs within NET series.

Looking to the future, the therapeutic landscape must evolve through international, multicenter prospective registries and pragmatically designed trials. Crucial to this paradigm shift will be dissecting the complex molecular landscape of lung NETs to identify novel oncogenic drivers and actionable therapeutic targets, spanning specific genetic mutations, epigenetic alterations, and aberrant signaling pathways. Concurrently, the development of next‐generation, highly selective compounds directed against these molecular vulnerabilities is paramount. In this scenario, future research should evaluate the role of chemotherapy both as a monotherapy and in combination with other modalities, including targeted therapies, RLT, and immunotherapy (such as novel ADCs and BiTEs targeting DLL3 and TROP2). Within this therapeutic landscape, future research must further clarify how targeted therapies as a whole, ranging from RLT to ADC‐ and BiTE‐based delivery, can be integrated or sequenced and investigate them within a broader precision medicine strategy aimed at addressing the specific molecular vulnerabilities of these malignancies.

**FIGURE 1:**
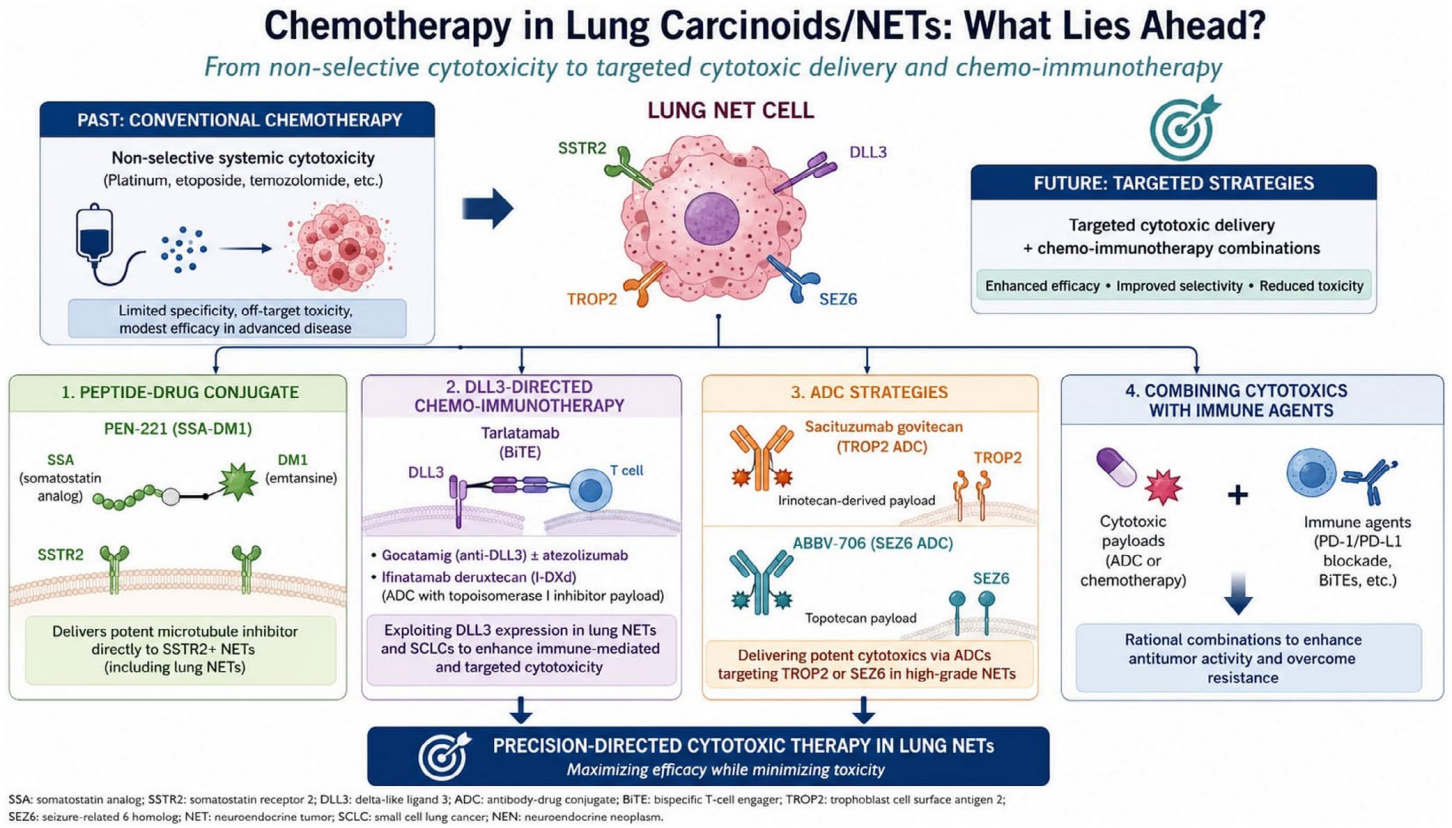
The evolving landscape of chemotherapy in lung NETs.

Ultimately, bridging the gap between historical retrospective limitations and future precision medicine will be the key to moving lung NET management from empirical, extrapolated choices to robust, evidence‐based, stage‐specific standards of care. In this context, Figure 1 serves as a conceptual model for potential future directions; readers should note that the integrated strategies shown remain largely theoretical for lung NETs, pending validation through the dedicated, histology‐specific trials mentioned above.

## Author Contributions

**Alice Laffi:** conceptualization, data curation, investigation, methodology, project administration, resources, supervision, validation, visualization, writing – original draft, writing – review and editing. **Anna La Salvia:** conceptualization, project administration, validation, visualization, writing – original draft, writing – review and editing. **Giuseppe Lamberti:** conceptualization, project administration, validation, visualization, writing – original draft, writing – review and editing.

## Funding

The authors have nothing to report.

## Ethics Statement

The study has been conducted in accordance with the Helsinki Declaration.

## Conflicts of Interest

The authors declare no conflicts of interest.

## Data Availability

Data sharing not applicable to this article as no datasets were generated or analyzed during the current study.
